# Lasting pathologic complete response to chemotherapy for ovarian cancer after receiving antimalarials for dermatomyositis

**DOI:** 10.3332/ecancer.2018.837

**Published:** 2018-05-22

**Authors:** Isabella Cadena, Victoria P Werth, Pascale Levine, Annie Yang, Andrea Downey, John Curtin, Franco Muggia

**Affiliations:** 1 New York University, New York, NY 10003, USA; 2 University of Pennsylvania, Philadelphia, PA 19104, USA

**Keywords:** ovarian cancer, dermatomyositis, antimalarials, autophagy, carboplatin sensitivity

## Abstract

Could hydroxychloroquine and quinacrine antimalarial therapy for dermatomyositis later attributed to a paraneoplasic manifestation of an ovarian cancer enhance its subsequent response to chemotherapy? Five months after being diagnosed with dermatomyositis, while somewhat improved with hydroxychloroquine, quinacrine and methotrexate, this 63-year-old woman presented with an advanced intra-abdominal epithelial ovarian cancer documented (but not resected) at laparotomy. Neoadjuvant carboplatin/paclitaxel resulted in remarkable improvement of symptoms, tumour markers and imaging findings leading to thorough cytoreductive surgery at completion of five cycles. No tumour was found in the resected omentum, gynaecologic organs, as well as hepatic and nodal sampling thus documenting a complete pathologic response; a subcutaneous port and an intraperitoneal (IP) catheter were placed for two cycles of IP cisplatin consolidation. She remains free of disease 3 years after such treatment and her dermatomyositis is in remission in the absence of any treatment. We discuss a possible role of autophagy in promoting tumour cell survival and chemoresistance that is potentially reversed by antimalarial drugs. Thus, chemotherapy following their use may subsequently lead to dramatic potentiation of anticancer treatment.

## Introduction

Surgical cytoreduction in stage IV epithelial ovarian carcinomas is of lesser efficacy relative to its key contribution in earlier presentations, presumably because the lower tumour burden decreases the likelihood of platinum resistance emerging during treatment [1]. Post-operative chemotherapy with carboplatin and paclitaxel has been the standard regimen even for most advanced presentations during more than two decades. With this doublet, up to 90% achieve objective responses, but the majority recur at a median of 18 months to 2 years, and in an analysis of cooperative group studies, only those undergoing R0 resections experience a superior 5-year survival exceeding 50% [2]. When primary surgical cytoreduction is not feasible, treatment shifts to neoadjuvant chemotherapy (NACT) followed by interval cytoreduction after 3–5 cycles. With either of these strategies, stage IV presentations have been associated with inferior results compared with stage III or earlier stages. This report documents complete pathologic response following NACT in a woman with stage IV ovarian cancer who prior to her cancer diagnosis had received several months of hydroxychloroquine and methotrexate, as well as quinacrine for dermatomyositis. After describing the highlights of her unusual presentation, we discuss whether her autoimmune disease and its treatment could have contributed to achieving such a striking therapeutic response.

## Case presentation

**Figure 1.**
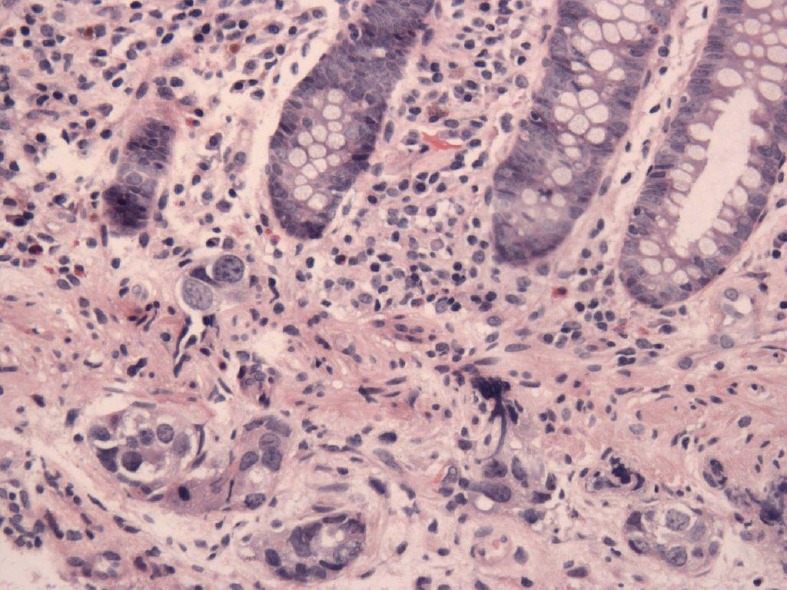
A submucosal protuberance in the transverse colon. Supplied by Dr Pascale Levine.

In April 2013, this 60-year-old mother of six children was diagnosed with dermatomyositis, with periungual telangiectasias, malar erythema, fatigue for the previous 6 months, and clinical as well as laboratory signs of muscle inflammation. Her initial treatment consisted of prednisone, and later continued with intermittent dexamethasone, hydroxychloroquine, quinacrine (added in June) and methotrexate (added in October). Additional medical history included chronic urinary complaints related to uterine prolapse since 1994. There was no family history of cancer or autoimmune disease. In November 2013, abdominal discomfort led to a colonoscopy with the finding of a submucosal protuberance in the transverse colon (Figure 1); biopsy was consistent with a poorly differentiated carcinoma and immunohistochemical features of a high-grade serous malignancy of likely gynaecologic origin. Further evaluation by computerised tomography (CT) identified abnormalities confirmed at a subsequent laparotomy in December 2013: diffuse metastases up to 2 cm in liver parenchyma as well as 3 cm mesenteric and retroperitoneal nodes, an omental mass infiltrating the transverse colon, bilateral tubo-ovarian masses and a 2-cm cul-de-sac nodule; adnexectomies and omental biopsies were performed with pathology confirming high-grade serous carcinoma arising from the right and left fallopian tubes (American Joint Commission on Cancer (AJCC) stage pt3bNxM1, Fédération Internationale de Gynécologie et d’Obstétrique (FIGO) stage IV b). She received five cycles of NACT with carboplatin and paclitaxel from January 2014 to April 2014 (Table 1) with excellent tolerance except with her urinary symptoms and pelvic pressure. After normalisation of serum CA125 and CT findings, in May 2014, she underwent an exploratory laparotomy that included negative frozen section of the liver, a completion hysterectomy and absence of tumour in biopsies of the cul-de-sac-omentum, residual right ovary, abominal and vesical wall, coupled with uterosacral ligament suspension, anterior and posterior pelvic floor repair, midurethral synthetic sling, cystoscopy and insertion of an indwelling catheter and IP port. Post-operatively, one IV and two IP doses of 40 mg/m^2^ cisplatin were given as consolidation with the last treatment taking place in July 2014. Post-treatment CT and removal of the IP port took place later in 2014 when she was asymptomatic. In June 2015, an episode of cutaneous Herpes zoster (shingles) was treated with famcyclovir by her local physician a few days after its onset, with full resolution except for mild post-herpetic neuralgia. Otherwise, she has experienced no symptoms, and continues to have near complete relief of urinary symptoms after her bladder suspension operation. A routine colonoscopy in November 2017 (by the same specialist who diagnosed her transverse colon metastasis) showed an absence of the previous findings, and she remains well 4 months later.

## Experimental pathology findings

Immunohistochemistry was performed on formalin fixed, paraffin-embedded, 5-μm transverse colon tissue sections. Unconjugated, polyclonal rabbit antimicrotubule-associated protein 1, light chain 3 beta (LC3B, Santa Cruz Biotechnology Cat# sc-28266, Lot# H115 RRID: AB_2137719) raised against the N-terminal region of human LC3B and mouse monoclonal anti-SQSTM1/p62 (Abcam Cat# ab-56416, Lot# GR245897-1, RRID: AB_945626) raised against full-length recombinant human protein, were used for immunohistochemistry as described by Martinet *et al* [3]. In brief, deparaffinised sections were subjected to antigen retrieval for LC3B and p62 which was performed using Cell Conditioner 1 (Tris-Borate-ethylenediaminotetraacetic acid pH 8.5) and Cell Conditioner 2 (Citrate pH 6.0) each for 36 minutes, respectively. LC3B was diluted 1:200 (1.0 μg/mL) and p62 diluted (10 μg/mL) in tris-buffered saline (25 mM Tris, 0.15 mM NaCL and pH 7.2) with 1% bovine serum albumin and incubated for 3 hours at 37°C. Primary antibody was detected using goat antirabbit or goat antimouse horseradish peroxidase conjugated multimer incubated for 8 minutes and the complex visualised with 3, 3 diaminobenzidene and enhanced with copper sulfate. Slides were washed in distilled water, counterstained with haematoxylin, dehydrated and mounted with permanent media. Negative controls consisted of diluent only tested with the study sections.

## Results

Giant autophagosomes—possible effects of hydrochloroquine inhibition of autophagy—were not seen. LC3B immunostaining was absent throughout, whereas strong diffuse cytoplasmic immunostaining for p62 was seen. LC3B expression from the activation of autophagy would have been expected in metastases beyond primary specimens [4]; on the other hand, the expression of p62 suggests ongoing autophagy at some point during tumour growth.

## Discussion

Ovarian cancer is the second most common gynaecological malignancy after endometrial cancer and the most common cause of gynaecological cancer deaths in the United States, since it is mostly diagnosed in stage III (intra-abdominal metastases) [5]. Six cycles of platinum-based chemotherapy contribute to 80–90% objective responses following the primary cytoreductive surgery, but relapses take place in the vast majority with the median progression free survival ranges from 12 to 18 months. Increasingly, NACT has been applied to those presenting with even more advanced disease, and noninferiority trials have justified such use when optimal surgical cytoreduction is not achievable, or there are compelling medical reasons for not proceeding with primary surgical cytoreduction [6]. Ovarian cancer can be associated with dermatomyositis as a paraneoplastic manifestation [7], and a diagnosis of dermatomyositis should prompt workup for such an occult malignancy.

Dermatomyositis, an idiopathic inflammatory myopathy, has various clinical manifestations, including muscle weakness, skin eruptions, interstitial pulmonary disease and polyarthritis [8]. Up to 90% of patients experience muscle weakness that is accompanied by skin manifestations in 50% to 60% [9]. The pathognomonic skin lesions are Gottron’s sign, Gottron’s papules and a heliotrope [10]. Muscle weakness is usually proximal and symmetrical, typically affecting the deltoids and hip flexors [11]. Pathology typically reveals muscle fiber necrosis, inflammatory cell infiltrate and signs of regeneration. In some cases, fibrosis can be found interspersed with muscle fibers, presumably from capillary injury causing microinfarcts [12].

The treatment for dermatomyositis includes hydroxychloroquine and other antimalarial drugs that have recently received attention in experimental models as potentiators of chemotherapy when authophagic pathways are activated. Autophagy, a cellular response to stress, is characterised by double membrane vesicles that engulf cytoplasmic organelles, and subsequently attain their degradation via lysosomes, thus providing the necessary energy for survival under conditions of nutrient depletion [13, 14]. Autophagy has been evoked as a mechanism for promoting cell survival during cancer development and growth [15–19], and also as a cause of resistance to chemotherapy that is reversed by chloroquine [20, 21]. Studying autophagy-induced cell death in ovarian cancer cell lines, Khurana *et al* [22] observed a striking effect of carboplatin chemosensitisation in resistant cell lines when quinacrine was added. This group subsequently provided evidence that quinacrine induces autophagy-mediated downregulation of the p62-Skp2 axis leading to upregulation of p21/27 independent of p53 [23]. The exposure of our patient to antimalarials preceded the treatment of her tumour; nevertheless, her pathologic CR to chemotherapy notwithstanding the late stage of her ovarian cancer at presentation raises questions as to whether such pretreatment played a remarkable potentiating role.

This report of such an unusual ‘responder’ may generate greater awareness of circumstances modulating autophagic cell death. Possibly also relevant is the presence of the autoimmune paraneoplastic syndrome that prompted the use of antimalarials. As in the case of anti-Yo antibodies [24], severe autoimmune manifestations typically are first triggered in the presence of relatively small tumour burdens. Such a heightened antitumour immunity may also have played a role in this patient’s course and response to chemotherapy. Additional studies are under discussion to pursue this hypothesis.

## Conclusion

A high-grade serous ovarian cancer patient presenting with an aggressive biology with intramural metastases to colon and to liver shortly after a diagnosis of dermatomyositis and treatment with antimalarials is now 3 years continuously free of both diseases after being treated with neoadjuvant carboplatin and paclitaxel followed by post-operative cisplatin consolidation. This raises the question: do autophagy and/or antitumour immunity play a role in achieving this remarkable result from chemotherapy after exposure to antimalarials?

## Figures and Tables

**Table 1.** Neoadjuvant carboplatin/divided dose paclitaxel followed by post-operative cisplatin consolidation.

Treatment (route) & cycle	Date	Doses (mg)	CA125 U/ml
Neoadjuvant		Carboplatin Paclitaxel	
(IV)1a	1/13/14	628, 164	152.3
(IV)1b	1/24	164	204.4
(IV)2a	2/4	628, 164	127.9
(IV)2b	2/14	164	61.7
(IV)3a	2/25	628, 164	33.3
(IV)3b	3/7	164	15.1
(IV)4a	3/19	523, 164	9.4
(IV)4b	3/28	164	7.7
(IV)5a	4/8/14	418, 164	6.5
(IV)5b	4/18	164	11.6
None	4/29		7.0
Surgery & IP port placed	5/17		9.0
Cisplatin postoperative			
(IV)cycle 1	6/6	67	10.9
(IP)cycle 2	7/1	67	6.8
(IP)cycle 3	7/29/14	68	5.7

IV = intravenous;

IP = intraperitoneal
